# From professional appeal to professional standard: reframing the Italian rehabilitation workforce through evidence, regulation, and global alignment

**DOI:** 10.3389/fpubh.2026.1797084

**Published:** 2026-05-04

**Authors:** Marco Tofani, Maria Ramiro-Gonzalez, Diego Poddighe, Justine Gosling, Federico Pennestrì, Antonella Cerchiari, Louise Puli, Carlotte Kiekens

**Affiliations:** 1 Management and Diagnostic Innovations & Clinical Pathways Research Area, Neurorehabilitation and Adapted Physical Activity Day Hospital, Bambino Gesù Children’s Hospital, IRCCS, Rome, Italy; 2 Department of Life Sciences, Health and Healthcare Professions, Università degli Studi “Link Campus University”, Rome, Italy; 3 Independent Consultant in Rehabilitation Workforce, Málaga, Spain; 4 Department of Rehabilitation Sciences, Research Group for Rehabilitation in Internal Disorders, KU Leuven, Leuven, Belgium; 5 University of Derby, Derby, United Kingdom; 6 Scientific Direction, IRCCS Galeazzi – Sant’Ambrogio Hospital, Milan, Italy; 7 PhD School in Neurosciences, Catholic University of Sacred Heart, Rome, Italy; 8 Rehabilitation, Ageing and Independent Living (RAIL) Research Centre, Monash University, Melbourne, VIC, Australia; 9 Laboratory of Evidence-Based Rehabilitation, IRCCS Galeazzi – Sant’Ambrogio Hospital, Milan, Italy

**Keywords:** education, health policy, healthcare economics and organizations, rehabilitation, standard of care, workforce

## Abstract

**Background:**

Italy’s rehabilitation workforce is fragmented and regulated by outdated decrees, limiting alignment with WHO and EU standards, and hindering efficiency, equity, and international comparability.

**Methodology:**

Narrative policy and health systems analysis, with comparative review of selected European models, based on secondary data and illustrative comparators.

**Policy issue:**

Workforce planning relies on historical professional categories and regional variability rather than population functioning needs and evidence-based competencies.

**Evidence:**

WHO Rehabilitation 2030 tools and European comparators show that coherent regulation, competency-based education, and need-driven planning improve service access, quality, and sustainability.

**Policy options:**

Align professional profiles with international standards; clarify scopes of practice; strengthen interprofessional education; integrate workforce planning with functioning and epidemiological data.

**Implications:**

Reform offers a strategic opportunity to reduce disparities, enhance system performance, and align Italy with global standards.

## 1 Introduction

Rehabilitation is a person-centered, goal-oriented, and collaborative process that aims to optimize an individual’s functioning and participation in society (1). It encompasses a wide range of interventions targeting both body structures and functions and environmental contextual factors influencing performance, with the ultimate purpose of enabling people with health conditions, or at risk of disability, to achieve and maintain the highest possible level of functioning (2). Rehabilitation is therefore an essential component of healthcare across the lifespan.

Globally, rehabilitation needs are substantial and growing. The Global Burden of Disease Study (2019) estimated that 2.41 billion people, about one in three worldwide, could benefit from rehabilitation services (3), with this burden further increasing in 2021, after the COVID-19 pandemic (4). This demand is driven by demographic ageing, the rising prevalence of chronic diseases, injuries, and non-communicable conditions, resulting in long-term functional limitations. Despite this, access to rehabilitation remains insufficient: more than half of the global population cannot obtain the services they need, with the greatest gaps in low- and middle-income settings (5, 6).

These global demographic and epidemiological transitions are particularly evident in high-income settings. Italy represents one of Europe’s oldest populations, with approximately 25% of the citizens aged 65 years or older. This demographic shift is linked to a rising prevalence of chronic conditions and long-term functional limitations (6, 7). According to the Organization for Economic Cooperation and Development (OECD), Italy has one of the highest life expectancies at birth among the members, and high quality healthcare workforce, on average; these positive connotations are counter-balanced by one of the highest functional limitation rates in patients older than 65 and persistent challenges in coordination and collaboration among healthcare professionals (8). Nearly 40% of the Italian population is affected by at least one chronic disease (9) and 30% of the patients of the Region of Lombardy, largely the most populated in Italy, accounts for 70% of the healthcare expenditure, due to chronic healthcare needs traditionally managed within a hospital system (10, 11). This demographic pressure further amplifies the need for a well-structured and sustainable rehabilitation workforce.

In response to these global challenges, the World Health Organization (WHO) has positioned rehabilitation as a fundamental component of Universal Health Coverage (UHC), launching the Rehabilitation 2030: A Call for Action in 2017 (12). This agenda was reinforced by the World Health Assembly Resolution WHA76 (2023), which urges the integration of rehabilitation into health system planning and financing, and highlights persistent shortages in both the number and quality of rehabilitation professionals (13). This global call underscores the urgent need for evidence-based workforce planning and investment in competency-based training, regulation and retention mechanisms to ensure equitable access. To support this process, the World Rehabilitation Alliance (WRA) was established in 2023 to foster evidence-based advocacy, policy dialogue, and multisectoral collaboration (14).

While WHO has articulated a global agenda for strengthening rehabilitation within health systems, implementation ultimately depends on national policy choices. Italy represents a relevant case study, as a comprehensive reform of health professions is currently under discussion at the Ministry of Health (MoH) (15). This reform provides a strategic opportunity to address long-standing regulatory fragmentation and regional variation in the rehabilitation workforce and rehabilitation services. If aligned with international standards and evidence-based frameworks, the ongoing reform could significantly strengthen rehabilitation workforce, and equitable service delivery.

Despite the growing recognition of rehabilitation as a core component of health systems, there is limited policy-oriented evidence examining how regulatory frameworks, education systems, and financing mechanisms interact to shape rehabilitation workforce composition and performance. In the Italian context, existing analyses have primarily focused on individual professions or service organization, with less attention to the systemic alignment between workforce governance, population needs, and international competency frameworks.

This paper aims to analyze the organization, financing, and workforce structure of rehabilitation in Italy, to assess their alignment with international standards and selected European models, and to identify evidence-informed policy recommendations to support workforce reform.

## 2 Methodology

This study employs a non-systematic narrative policy and health systems analysis to evaluate Italy’s rehabilitation service delivery, financing, and workforce governance against international benchmarks. The analysis is framed by the WHO Health Systems Framework and the International Classification of Functioning, Disability and Health (ICF), which provide a structured lens for examining rehabilitation needs, service organization, and workforce competencies.

Data were gathered through a triangulated search strategy encompassing: (1) global and regional policy and regulatory frameworks from the WHO and European Union; (2) Italian national legislation governing rehabilitation services and health professions; and (3) a targeted review of peer-reviewed literature. The literature review focused on policy documents, regulatory frameworks, and peer-reviewed studies addressing rehabilitation workforce, health system organization, and financing. Sources were selected based on relevance to the study objectives and alignment with WHO and European policy frameworks. No formal inclusion or exclusion criteria were applied, consistent with the narrative policy analysis approach.

A descriptive comparative analysis was conducted using Belgium and Spain, selected as illustrative European comparators due to their differing governance and financing models, as well as the availability of structured policy and organizational data relevant to rehabilitation services. Findings were synthesized using a thematic comparative approach across four domains: service delivery, financing, workforce regulation, and governance, enabling structured comparison between countries and identification of policy-relevant patterns.

As a narrative policy analysis, this study is based on a selective synthesis of relevant sources and is not intended to provide exhaustive coverage of literature. Ethical approval was not required, as the study relied exclusively on publicly available secondary data.

## 3 Rehabilitation delivery and financing

Effective rehabilitation requires coordinated service delivery across the continuum of care, spanning acute hospital settings, post-acute services, and community- and home-based settings. International evidence shows that fragmented or hospital-centered models are associated with inefficiencies, prolonged hospital stays, and suboptimal functional outcomes (16–18). Consequently, many health systems are shifting toward integrated models that prioritize early intervention at the primary level of care, continuity of care, and multidisciplinary collaboration.

While the pyramidal model of rehabilitation within health systems is useful for organizing rehabilitation services by care intensity (14), contemporary rehabilitation frameworks increasingly emphasize transversal, person-centered, and integrated models that deliver rehabilitation across settings, levels of care, and the life course, including outside the health sector.

**Figure 1:**
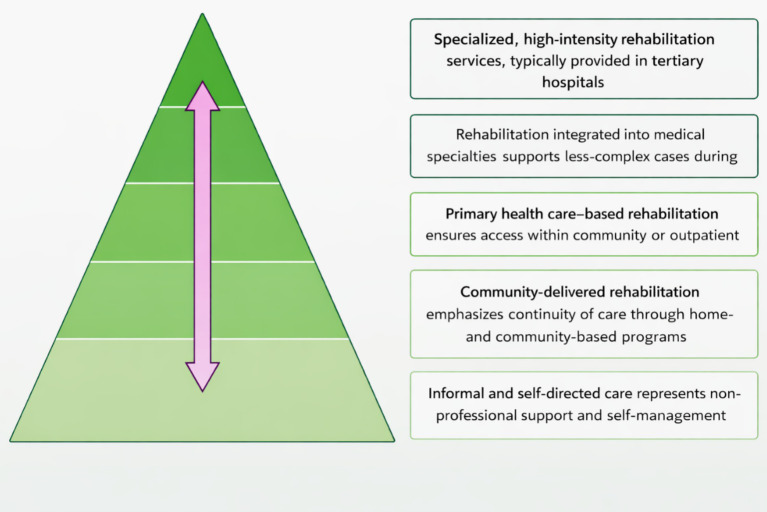
Organization of rehabilitation services within the WHO framework.

The WHO framework for rehabilitation within health systems is organized as a pyramid reflecting care intensity and setting (14). Specialized, high-intensity services for complex needs are delivered at the tertiary level, while decreasing complexity corresponds to integration into medical specialties, primary care and community settings. Home- and community-based rehabilitation promotes continuity of care and informal, self-directed activities from the base of the pyramid (Figure 1).

Financing mechanisms strongly influence rehabilitation delivery and workforce organization. Traditional payment models, including per diem reimbursement and Diagnosis-Related Groups (DRGs), were designed to enhance hospital efficiency, but they often incentivize service volume rather than continuity and functional recovery when applied to rehabilitation. DRG-based reimbursement may encourage premature discharge and “cream skimming,” contributing to fragmented care pathways, preventable complications, and higher costs (17–19). In contrast, bundled and value-based payment models that link reimbursement to continuity of care and functional outcomes have shown potential to improve efficiency, access, and quality, although their scalability and long-term sustainability require further evaluation (20–23).

Sustainable and equitable rehabilitation financing should be guided by population health needs, demographic trends, and functional outcome data, rather than historical expenditure patterns. In this context, functioning frameworks such as the ICF (24) are increasingly recognized as essential tools to align financing, service delivery, and outcome monitoring. A comparative overview of rehabilitation financing models is provided in Table 1.

**Table 1:** Comparative overview of rehabilitation financing models.

Payment model and description	Incentives and implications for rehabilitation	Key strengths	Main challenges/limitations
Per diem (per-day payment)			
Fixed daily rate paid for each inpatient day, regardless of services provided.	Incentivizes longer inpatient stays to maximize revenue, with limited motivation for efficiency. In rehabilitation, this may prolong length of stay without corresponding improvements in functional outcomes and weaken incentives for discharge planning and care transitions.	Simple to administer; predictable revenue stream for providers.	Can increase overall costs; encourages unnecessary length of stay; does not reward quality or outcomes.
Diagnosis-related group (DRG)			
Fixed, case-mix–adjusted payment per admission based on diagnosis and expected resource use.	Incentivizes shorter stays and cost control within the acute episode. In rehabilitation, this can result in early discharge or “cream skimming” of less complex cases, with limited alignment with goals of continuity and long-term functional recovery.	Promotes efficiency and standardization within hospitals; enables benchmarking.	Risks of premature discharge; poor continuity of post-acute care; limited focus on long-term functioning.
Bundled payment (episode-based)			
Single, fixed payment covering all services across a defined episode of care (e.g., acute, post-acute, and community rehabilitation).	Encourages coordination across providers and settings, reducing duplication and fragmentation. For rehabilitation, this supports continuity of care and shared accountability for functional outcomes across the care pathway.	Promotes integrated care and cost control across the continuum.	Complex to design and implement; requires strong data systems and clear outcome attribution; potential disputes over payment distribution.
Value-based payment (VBP)			
Payment partly or fully linked to achievement of predefined outcomes (e.g., functional improvement, patient satisfaction, reduced readmissions).	Rewards quality and outcomes rather than volume. In rehabilitation, it incentivizes patient-centered goals, measurable functional gains, and alignment between financing and rehabilitation objectives.	Links funding to performance; drives quality improvement and accountability.	Requires robust outcome measurement, risk adjustment, and appropriate data infrastructure; long-term sustainability yet to be proven.

In Italy, the introduction of DRGs contributed to improving hospital efficiency and standardization by linking reimbursement to case-mix and activity (25). However, the extent to which this mechanism has enabled effective competition between public and private providers remains limited, both for structural reasons inherent to the system and for incomplete implementation over time (25, 26).

Alternative financing models, such as bundled or value-based payments, have not been systematically adopted at the national level. Some regional experiences provide partial but informative insights. In Lombardy, between 2015 and 2018, chronic care management strategies introduced elements of pathway-based financing, including the assignment of a care manager responsible for coordinating services along individualized care pathways and additional remuneration linked to pathway management. While these initiatives aimed to improve coordination and accountability across providers, their implementation was interrupted by the COVID-19 pandemic (19) and subsequent primary care reforms (10), limiting the possibility of a robust evaluation of their impact.

More recently, value-based initiatives incorporating patient-reported outcome measures (PROMs) have been described in Italy by the Organization for Economic Cooperation and Development, reporting improvements in functional outcomes and quality of life following major joint replacement (OECD Health Working Papers No. 148). However, these initiatives remain limited to specific clinical areas and are not yet structurally linked to reimbursement mechanisms or workforce planning.

Taken together, these experiences suggest that financing reforms in Italy have been implemented mainly at the level of service organization, with limited attention to their implications for workforce configuration. This is particularly relevant as reimbursement models are not neutral with respect to workforce configuration. Payment mechanisms influence which professional competencies are valued, how teams are structured, and whether task-shifting or advanced practice roles are incentivized or constrained (27, 28). For instance, per diem or hospital-centered reimbursement may reinforce medically dominated models and limit the deployment of community-based or autonomy-driven rehabilitation professionals. Conversely, bundled or value-based payments can create incentives for coordinated multidisciplinary teams, clearer skill mix allocation, and outcome-oriented practice (28, 29). Some pilot experiments in the Italian regions have demonstrated for example that a multidisciplinary management of the chronic patient - both through the cooperation between general practitioner, community nurse and rehabilitation professionals, in the Tuscany Region (DGR Region of Tuscany 716/2019) (30) and through the use of a forfeit monetary budget in charge to the general practitioner, in the Lombardy Region (31)–lead to a better therapeutic adherence (DGR Region of Tuscany 1152/2015, Allegato A), a reduction in the mortality rate, a reduction in hospitalizations and inappropriate access to the emergency room (32). Therefore, financing reform and workforce reform should be considered interdependent dimensions of system redesign rather than parallel processes.

## 4 Rehabilitation workforce

The rehabilitation workforce is the cornerstone of service delivery (33). The composition of the rehabilitation workforce can vary between countries and settings; however, the competencies required to deliver rehabilitation are generally represented within the professions of audiology (AUD), clinical psychology, occupational therapy (OT), prosthetics and orthotics (P&O), physiotherapy (PT), and speech and language therapy (SLT), as well as by physical and rehabilitation medicine doctors (PRM) and nursing (34, 35). In addition, the rehabilitation workforce often includes assistants, technicians and community-based rehabilitation workers (36, 37).

Globally, the rehabilitation workforce is affected by persistent shortages, uneven geographic distribution, and limited integration within health systems (38, 39). In many low- and middle-income settings, fewer than 10 rehabilitation professionals are available per million population (12), highlighting a critical gap in service capacity. Marked disparities also exist across regions: in the WHO European Region the shortage is particularly acute in the 21 middle-income countries, where, in 2016, the number of rehabilitation professionals was substantially lower, with 12 times fewer PT, 141 times fewer OT, 6 times fewer P&O, and 3 times fewer PRM practitioners than in the 32 high-income countries of the Region (40). These disparities are not solely related to workforce supply, but reflect differences in regulatory frameworks, education systems, and health system organization. Together these factors influence workforce production, distribution, and utilization across settings. These structural factors, in turn, shape how the workforce is planned, trained, and deployed within health systems. Inadequate workforce planning, insufficient training opportunities, and limited career pathways exacerbate these challenges. Addressing these gaps requires comprehensive strategies beyond increasing workforce numbers, including strengthening competencies, ensuring equitable access, fostering multidisciplinary collaboration, and enhancing professional recognition and leadership (41).

Beyond regulatory alignment, it is important to recognize the evolving role of rehabilitation professionals in contemporary health systems. In several countries, OT, PT and SLT have expanded their scope through advanced practice roles, direct access pathways, and structured triage models within primary and community care (42, 43). These developments illustrate how clearly defined competencies, supported by coherent regulatory frameworks, can enhance service responsiveness, reduce care delays, and strengthen integration across levels of care (44, 45). Positioning rehabilitation professionals as drivers of service innovation may therefore represent not only a regulatory adjustment but a strategic opportunity for system modernization.

The expansion of professional roles and responsibilities, however, requires robust governance mechanisms, standardized competency frameworks, and sustained investment in education and regulation. In this regard, the WRA plays a key role in advancing global advocacy for the rehabilitation workforce. Through its Workforce Workstream, the WRA promotes investment in high-quality education and training as a foundation for expanding equitable access to rehabilitation. It calls upon Member States to strengthen university programs, develop competency-based curricula, ensure continuous professional development, and foster partnerships across ministries, academic institutions, professional associations, and other rehabilitation stakeholders (46).

## 5 Rehabilitation in Italy: organization, services, and policy framework

Rehabilitation in Italy is an integral component of the Servizio Sanitario Nazionale (SSN), a Beveridge-type health system funded through general taxation that guarantees universal access to care. Rehabilitation is formally included among Essential Levels of Care (LEA), which define the services that all Regions must provide nationwide (47, 48). Within this framework, rehabilitation is recognized as a core dimension of healthcare, and addresses acute, post-acute, and chronic conditions, long-term disability and functional decline.

A key milestone was the adoption of the National Rehabilitation Plan in 2011 (49, 50), which provides national guidance for the development and coordination of rehabilitation services, promoting continuity of care, person-centered approaches, and multidisciplinary delivery. Governance is highly decentralized: while the MoH defines the national strategic framework, regions are responsible for planning, financing, and service delivery. This structure allows contextual adaptation but also results in substantial regional variability in service availability, eligibility criteria, and access (48). Within this governance framework, the Italian National Institute of Health (ISS) plays a central role in the development and coordination of national clinical guidelines through the National Guideline System (Sistema Nazionale Linee Guida – SNLG). Under the mandate of the MoH, the ISS is responsible for methodological oversight, accreditation of guideline-producing bodies, and the appraisal of clinical recommendations, including those relevant to rehabilitation. However, the number of rehabilitation-specific guidelines formally developed and validated within the SNLG remains limited, and guideline implementation is not systematically linked to financing or workforce planning mechanisms. As a result, the translation of evidence into practice and the standardization of rehabilitation pathways remain uneven across the country. Rehabilitation is delivered through a diversified network of hospital-based units, inpatient post-acute facilities, outpatient and day-hospital services, residential structures, community-based programs, and home care, provided by both public and accredited private providers. Recently, the Rehabilitation Hospital Discharge Form (49) was introduced and represents an important step toward improved data collection, appropriateness of referral, and continuity across care settings (51). However, their implementation remains uneven across the country.

Rehabilitation financing is primarily tax-based, with resources allocated to regions. Inpatient rehabilitation is commonly reimbursed through per diem payment models (Table 1), often combined with expenditure caps. Outpatient and community-based services may involve co-payments (ticket), with exemptions for children, people with chronic conditions, low-income households, and people with disabilities.

AT is included within the LEA and regulated through the *Nomenclatore Tariffario* (D.M. 332/1999 and subsequent updates), which defines eligibility and reimbursement criteria. Despite a national regulatory framework, access to AT remains regionally variable, particularly regarding authorization procedures, delivery timelines, and availability of advanced or customized devices.

Overall, the system ensures broad entitlement to rehabilitation and AT and provides substantial financial protection against catastrophic expenditure. However, high regional autonomy–often compounded by financial recovery plans imposing expenditure and workforce constraints – has generated persistent disparities in access, organization, and quality. Rehabilitation financing remains fragmented, largely hospital-centered, and weakly linked to outcomes, with planning frequently based on historical utilization rather than population needs or epidemiological projections (52).

Different models of rehabilitation service delivery reflect varying approaches to governance, financing, and the integration of evidence-based practice. Comparative experiences from Belgium and Spain–both characterized by a mature rehabilitation system and substantial differences in governance and performance monitoring–illustrate different approaches adopted within European health systems (17, 50, 53). These models highlight how different balances between central regulation, regional autonomy, and performance monitoring can shape rehabilitation service organization and provide relevant insights for Italy’s ongoing health reform (Table 2).

**Table 2:** Comparison across three different models for rehabilitation delivery in Europe.

Domain	Italy	Belgium	Spain	Comparative insights
Model type	Beveridge-type National Health Service (SSN), publicly financed, universal entitlement.	Bismarck-type Social Health Insurance system, compulsory for all citizens, managed through mutual insurance funds (*mutualités*).	Beveridge-type National Health System (Sistema Nacional de Salud – SNS), publicly financed, universal coverage.	Italy/Spain: tax-funded Beveridge systems with decentralized governance. Belgium: compulsory social health insurance Bismarck system. Different financing models, but governance mechanisms are relevant for rehabilitation coherence.
Financing	Funded mainly by general taxation; resources allocated to Regions. Rehabilitation and assistive technology included within the *Livelli Essenziali di Assistenza* (LEA).	Funded through social health insurance contributions; centralized financing via *RIZIV/INAMI* nomenclature and conventions. Parts of long-term care and assistive technology are funded by the Regions.	Funded mainly through general taxation allocated to regional governments. Rehabilitation services are included in the common benefits package of the SNS (Cartera Común de Servicios). Some co-payments apply for some orthopaedic prostheses and assistive products.	Italy/Spain: decentralized allocation of tax-funded budgets; greater regional variation. Belgium: centralized financing through RIZIV/INAMI conventions. Centralized financing frameworks could support financial consistency in Italy.
Service delivery	Multi-level structure: (acute, post-acute, and long-term rehabilitation). Specialized rehab units in hospitals. Community settings, and home-based care.	Multi-level structure: (acute, post-acute, and long-term rehabilitation). Specialized rehab units in hospitals. Community settings, and home-based care.	Multi-level structure: acute, post-acute, and long-term rehabilitation. Specialized rehabilitation departments in hospitals and reference centers. Rehabilitation units in community settings, and home-based care.	All three countries: multi-level rehabilitation structure (acute, post-acute, community). Differences in rehabilitation systems relate more to governance and financing than to service delivery structure.
Service organization	Highly regionalized system under national coordination of Ministry of Health. Providers for both rehabilitation and assistive technology accredited by the regions and monitored by local healthcare units	Centrally organized through national conventions defining structure, personnel, and reimbursement. Providers accredited and monitored by *RIZIV/INAMI*. Parts of long-term care and assistive technology are organized by the Regions.	Highly decentralized system: regional health services organize and provide rehabilitation. The Ministry of Health coordinates national strategies, benefit packages, and interregional cooperation through the Interterritorial Council of the SNS.	Italy/Spain: mainly regional regulation (Spain with national coordination mechanisms). Belgium: national organization through conventions with partial regional implementation. A combined national–regional organizational model may improve harmonization of rehabilitation services.
Number and duration of rehabilitation sessions	Minimum intensity classified as high, medium, or low; Inpatient rehabilitation depending on DRG no national limit on number of sessions. Duration depends on patient needs and clinical justification.	Defined in nomenclature and *RIZIV/INAMI* conventions; number of sessions predetermined per condition (e.g., stroke or SCI).	Not nationally standardized. The number and duration of rehabilitation sessions are determined by clinical assessment.	Italy/Spain: flexible clinical prescription. Belgium: condition-specific predetermined. Structure mechanisms may support transparency and comparability.
Evidence-based framework and reimbursement	*Diagnosis Related Groups (DRG)* and per diem tariffs defined by the intensity of care (high, medium, or low). Outpatient rehabilitation follows a fee-for-service model with possible co-payments. Although the *Bianco-Gelli Law* (2017) mandates adherence to evidence-based clinical guidelines, the National Guideline System (*SNLG* – Istituto Superiore di Sanità) currently provides only limited rehabilitation-specific guidelines. Outcome or indicators are not linked with reimbursement.	Reimbursement is based on fixed national tariffs established through *RIZIV/INAMI* conventions and pathology-specific classifications. Evidence-based protocols and quality indicators are embedded in the accreditation process and monitored at the national level, ensuring standardized care delivery; however, payments remain case- or session-based and are not directly linked to patient outcomes	Hospital care uses Diagnosis Related Groups (DRG)-based financing for inpatient activity, while outpatient rehabilitation is funded through regional budgets and service contracts. Clinical practice guidelines and national health strategies promote evidence-based care; however, functional outcomes or quality indicators are not directly linked to reimbursement.	Italy/Spain: clinical guidelines not linked to financing. Belgium: protocols and indicators linked to accreditation and monitoring, but not to financing. Integrating guidelines with quality monitoring may strengthen evaluation of rehabilitation services.

Overall, while all three countries share a multi-level structure for rehabilitation service delivery, differences in governance and financing mechanisms appear to influence system coherence and performance (14, 17). In particular, more centralized regulatory and financing frameworks, as observed in Belgium, which is a small but complex federal state, may support greater consistency in service provision and monitoring, whereas unitary but highly decentralized systems, such as Italy and Spain, are associated with increased regional variability in access and organization (7, 17). Although standardized performance indicators are not systematically available across countries, comparative evidence suggests that system performance can be interpreted in terms of consistency of service provision, integration across care levels, and alignment between financing mechanisms and functional outcomes (16, 17). In this perspective, the Italian system appears particularly affected by fragmentation between regulatory frameworks, financing mechanisms, and service organization, which may limit efficiency, equity, and continuity of care compared to more coordinated models.

## 6 Rehabilitation workforce in Italy: organization and challenges

The governance of health professions in Italy is organized within a clearly defined legislative hierarchy. Law 10 August 2000, n. 251 formally recognized the autonomy and professional responsibility of non-medical health professions within their respective areas of competence. Law 11 January 2018, n. 3 subsequently strengthened the institutional role of Professional Orders and national federations, consolidating their regulatory functions in matters of registration, professional standards, and ethical oversight under the supervision of the MoH.

While physicians, nurses and midwives are represented by their respective national professional orders, PTs are the only rehabilitation profession represented by a dedicated national federation (FNOFI – Italian Federation of Physiotherapists). All other rehabilitation professions are integrated into a multi-professional body, the Federazione Nazionale degli Ordini TSRM e delle Professioni Sanitarie Tecniche, della Riabilitazione e della Prevenzione (FNO TSRM-PSTRP), which encompasses technical rehabilitation and preventive health professions.

The national framework for university education and professional qualification in health professions was established through the Interministerial Decree of 19 February 2009. Within this framework, non-medical health professions are organized into degree classes grouping occupations with comparable functions, competencies, and scopes of practice, while profession-specific profiles are defined by individual Ministerial Decrees. Table 3 presents the current degree class, associated professions, and corresponding professional national federation. PRM are not included in these degree classes, as they follow the medical education pathway, consisting of a six-year medical degree followed by four-year postgraduate specialty training.

**Table 3:** Organization of healthcare professions in Italy.

Degree class	Health profession	Ministerial decree (D.M.)	Federation
L/SNT1–Nursing and midwifery professions	Nurse	D.M. 14 September 1994, No. 739	FNOPI
	Pediatric nurse	D.M. 17 January 1997, No. 70	FNOPI
	Midwife	D.M. 14 September 1994, No. 740	FNOPO
L/SNT2–Rehabilitation professions	Physiotherapist	D.M. 14 September 1994, No. 741	FNOFI
	Speech and language therapist	D.M. 14 September 1994, No. 742	FNO TSRM-PSTRP
	Occupational therapist	D.M. 17 January 1997, No. 136	FNO TSRM-PSTRP
	Neuro and psychomotor therapist of developmental age	D.M. 17 January 1997, No. 56	FNO TSRM-PSTRP
	Psychiatric rehabilitation technician	D.M. 29 March 2001, No. 182	FNO TSRM-PSTRP
	Professional educator	D.M. 8 October 1998, No. 520	FNO TSRM-PSTRP
	Orthoptist and ophthalmology assistant	D.M. 14 September 1994, No. 743	FNO TSRM-PSTRP
	Podiatrist	D.M. 14 September 1994, No. 666	FNO TSRM-PSTRP
L/SNT3–Technical health professions	Medical radiology technician	D.M. 26 September 1994, No. 746	FNO TSRM-PSTRP
	Biomedical laboratory technician	D.M. 14 September 1994, No. 745	FNO TSRM-PSTRP
	Audiometrist technician	D.M. 14 September 1994, No. 667	FNO TSRM-PSTRP
	Audiologist technician	D.M. 14 September 1994, No. 668	FNO TSRM-PSTRP
	Prosthetics and orthotics technicians	D.M. 14 September 1994, No. 665	FNO TSRM-PSTRP
	Neurophysiopathology technician	D.M. 15 March 1995, No. 183	FNO TSRM-PSTRP
	Cardiovascular perfusion technician	D.M. 27 July 1998, No. 316	FNO TSRM-PSTRP
	Dental hygienist	D.M. 15 March 1999, No. 137	FNO TSRM-PSTRP
L/SNT4–Preventive health professions	Health assistant	D.M. 17 January 1997, No. 69	FNO TSRM-PSTRP
	Technicians for prevention in the Environment and in the Workplace	D.M. 17 January 1997, No. 58	FNO TSRM-PSTRP
	Osteopath*	Presidential Decree 7 July 2021, n. 131	

FNOPI: Italian Federation of Nursing Professions; FNOPO: Italian Federation of Midwives; FNOFI: Italian Federation of Physiotherapists; FNO TSRM-PSTRP: Italian Federation of Health Technical, Rehabilitation and Prevention Professions. *Osteopaths have been recently recognized as a new healthcare profession within the L/SNT4 degree class. According to the current legislation, osteopaths are primarily authorized to perform preventive and health maintenance activities through manual treatment of somatic dysfunctions, rather than clinical rehabilitation. Their integration within the healthcare system is still under discussion. However, osteopaths are expected to be included in the FNO TSRM-PSTRP once the regulatory process is completed.

Within the constitutional framework of the SSN, however, responsibility for the organization and delivery of health services is largely entrusted to the regions. This separation between nationally defined professional scopes and regionally managed service organizations introduces structural complexity into the implementation of competencies. Reforms aimed at clarifying or redefining scopes of practice must operate within a multilevel legal architecture in which legislative provisions, regional authority, and professional self-regulation intersect. Scope-of-practice reform is therefore not simply a technical revision of professional boundaries, but a process embedded in constitutional and institutional arrangements. In this context, the regulatory structure, while historically grounded, contributes to fragmentation by reinforcing profession-based classifications rather than competency-based organization of care, potentially limiting flexibility in workforce planning and integration across services.

These degree classes correspond to Level 6 of the European Qualifications Framework (EQF) and consist of three-year Bachelor programs (180 CFU) with substantial supervised clinical training, followed by access to postgraduate education (postgraduate certificates, Master’s, and PhD programs). While formally aligned with the Bologna Process and EU Directive 2005/36/EC, substantial cross-country differences persist in educational standards, competency profiles, and professional recognition. In some EU Member States, comparable rehabilitation qualifications are assigned to higher EQF levels (54), limiting automatic recognition of Italian degrees and often requiring compensatory measures for professional mobility. While the Bologna Process established a shared higher education architecture, it did not define minimum competency standards for rehabilitation practice. As highlighted in the 2018 EQF report (54), many National Qualification Frameworks prioritized formal qualification levels over competency alignment, a gap that ongoing Italian reforms are now expected to address.

Within this educational and regulatory framework, the Italian rehabilitation workforce is characterized by a historically stratified and administratively fragmented classification. This structural fragmentation has measurable consequences: empirical evidence from recent national analyses indicates that workforce fragmentation in Italy is associated with uneven geographic distribution, supply shortages, and misalignment between workforce capacity and population needs, particularly in rehabilitation services (49).

To contextualize these findings, international classifications provide a useful reference. Globally, PT, OT, SLT are universally recognized as core rehabilitation professions and are represented by global professional federations such as World Physiotherapy (WP), World Federation of Occupational Therapists (WFOT), and International Association of Communication Sciences and Disorders (IALP). However, some professions that are internationally considered integral to rehabilitation–most notably P&O and AUD – are formally classified under the technical health professions instead of the rehabilitation professions, reflecting historical regulatory decisions rather than differences in competencies, or populations served. Further complexity arises from the presence of several professions that are specific to the Italian context, including Neuro-and Psychomotor Therapists of Developmental Age (TNPEE), Psychiatric Rehabilitation Technicians (TERP), and Professional Educators (EP). These professions lack a direct equivalent in other countries and exhibit partially overlapping scopes of practice with other rehabilitation professionals. This situation complicates workforce governance, and limits professional mobility and international collaboration.

Institutional classification also carries operational consequences. In Italy, training positions for health professions are closely linked to formal professional categories and to requests emerging from the healthcare system itself (55). As documented in analyses of Italian health workforce governance (52, 56) planning has largely been conducted as a demographic replacement exercise, primarily based on age structure and retirements forecasts rather than on systematic assessment of population health needs or competency requirements. This approach tends to reproduce historically established professional configurations. When educational quotas are defined within pre-existing regulatory categories, the flexibility to realign and to prioritize workforce composition in response to epidemiological transitions, functional needs, emergencies, or emerging models of care become limited. This feedback loop between regulatory classification and educational planning contributes to maintaining legacy professional boundaries rather than facilitating competency-based restructuring (52). A recent systematic review (57) concluded that health workforce planning approaches are not fully anchored to major transformations in healthcare systems, suggesting the persistence of incremental and profession-centered models internationally. Within this broader methodological context, the Italian case reflects a planning approach in which regulatory categories and demographic projections have played a more prominent role than systematic alignment with evolving population needs and service models. From a policy perspective, this configuration may limit the ability of the system to align workforce competencies with population needs, constraining efficiency, integration, and responsiveness of rehabilitation services.

## 7 Discussion: gaps, opportunities, and policy implications

Italy is entering a decisive phase in the restructuring of its rehabilitation workforce. The long-awaited reform of health professions currently under discussion (15) represents an opportunity to align and modernize the Italian rehabilitation system with international standards for population benefit. To succeed, the reform must address long-standing challenges in governance, education, and professional integration that limit system coherence and workforce effectiveness.

### 7.1 Blurred professional boundaries and regulatory misalignment

A central weakness concerns the heterogeneity and misalignment among rehabilitation professions. Compared with other EU countries, Italy retains a fragmented set of rehabilitation profiles, many still defined by decrees from the 1990s rather than contemporary evidence on effective and safe interventions or international competency and regulatory standards. While professional profiles, clinical guidelines and good practices should align workforce competencies with scientific evidence, in Italy outdated regulatory frameworks have instead generated overlapping scopes of practice, blurred professional boundaries, and inconsistent educational standards (52, 58–60). This reflects broader European evidence showing that historically layered regulatory frameworks contribute to role ambiguity and skill mismatches (61). As a result, weak governance of scopes of practice and competency frameworks (62) can lead distinct professions to deliver similar interventions within the same functional domains, reflecting role blurring and overlapping tasks observed in clinical settings (52, 63), and contributing to challenges in accountability, workforce efficiency, equity and quality of rehabilitation care (64).

Regulatory misalignment produces inefficiencies through identifiable causal mechanisms. When professional profiles are defined by outdated or overlapping decrees, scopes of practice become blurred, leading to duplication of functions and unclear accountability within teams (34, 64). The absence of clearly articulated competency frameworks complicates workforce planning, as educational quotas and staffing standards cannot be coherently aligned with defined competencies (56, 62). Service delivery may rely on historically established role distributions rather than population needs or evidence-based intervention models (56, 65). Consistently, international analyses report skill mismatch, fragmented distribution, and limited integration as consequences of weak regulatory alignment (37, 38). At system level, insufficient coordination between regulatory frameworks, competency standards, and workforce planning undermines the development of coordinated and outcome-oriented service delivery (66).

### 7.2 Educational and professional standards misalignment

A further critical issue is the limited harmonization of Italian professional and educational frameworks with European and international standards. Despite Italy’s adherence to the Bologna Process (67) and to EU Directive 2005/36/EC on the recognition of professional qualifications, many rehabilitation programs remain misaligned with the competency frameworks of global federations such as WP, the WFOT, IALP, and the International Society for Prosthetics and Orthotics (ISPO). Professions internationally recognized within rehabilitation, such as P&O and AUD, are instead classified in Italy under the “technical” category, struggling to find proper collocation in the healthcare system, while several uniquely Italian professions (e.g., TNPEE, TERP, EP) lack international equivalents, restricting professional mobility and comparability of workforce data.

These inconsistencies reflect broader challenges in aligning workforce education with evolving health system needs. Evidence from health workforce governance research indicates that education and training systems frequently remain structured around historically defined professional categories rather than shared competencies or population health needs (65). As a result, training pipelines may reproduce existing occupational structures even where scopes of practice overlap or where contemporary models of care require more flexible skill-mix configurations (68). This limits the development of integrated and interprofessional models and reduces system adaptability to demographic and epidemiological change (69). Aligning educational standards with internationally recognized competency frameworks and collaborative practice models is therefore increasingly considered a key component of effective health workforce governance (65, 68).

### 7.3 Structural workforce planning limitations

A third major limitation concerns the absence of a comprehensive, evidence-based workforce planning strategy. Educational quotas are often set through supply-driven approaches, with limited systematic reference to population needs, epidemiological projections, or service coverage indicators (65). In Italy, the lack of integrated national monitoring systems, capable of linking workforce supply, geographic distribution and rehabilitation service utilization, limits the timely identification of shortages, regional disparities, and emerging skill needs. For example, data on the distribution of rehabilitation professionals are not routinely connected to indicators of population functioning or service demand; consequently, rehabilitation workforce planning remains largely reactive and fragmented, driven by historical training patterns rather than measurable population needs or system priorities (52, 58).

Overall, these issues reflect the interaction between regulatory misalignment and structural weakness in workforce governance. The persistence of supply-driven educational quotas, the absence of integrated national monitoring systems linking workforce supply with service utilization and population functioning indicators, and the limited use of epidemiological projections or service coverage data have constrained the development of evidence-based workforce strategies. As a result, workforce configuration tends to reproduce historically established professional categories rather than adapt dynamically to evolving population needs and models of care.

The reform represents an opportunity to strengthen rehabilitation and, in doing so, promote the principles of equity, efficiency, and transparency. Workforce planning should use population health indicators, functioning data, and epidemiological trends (70, 71), in line with WHO recommendations for needs-based health workforce strategies (66, 72, 73). Developing evidence-based national policies and guidelines is essential to ensure that workforce composition reflects the best available evidence on effective interventions and models of care. In practice, this requires profession-specific guidelines–developed and appraised through robust methodologies such as Grading of Recommendations Assessment, Development and Evaluation (GRADE)–to inform decisions on workforce mix, competences, and service delivery based on patients’ case-mix and needs. Where profession-specific evidence is lacking, competency-based frameworks and intervention-level evidence should identify which cadres possess the skills required to deliver effective, safe, and efficient rehabilitation.

Importantly, these challenges offer a strategic opportunity for systemic modernization. The suite of WHO tools: Rehabilitation 2030 (12), the Package of Interventions for Rehabilitation (PIR) (36, 74), the Rehabilitation Competency Framework (RCF) (34), and the Guide for Rehabilitation Workforce Evaluation (GROWE) (66), provides an evidence-based roadmap for countries to strengthen their rehabilitation sectors. Through the systematic use of these instruments, Italy could assess current workforce capacity, identify skill mismatches, and align educational standards with internationally recognized professional profiles. Likewise, the Systematic Assessment of Rehabilitation Situation (STARS) framework (73) offers a data-driven method for evaluating service coverage and unmet needs, guiding targeted investments and informed policy decisions.

## 8 Conclusion and recommendations

Italy stands at a pivotal crossroads. Reform of the rehabilitation workforce—grounded in functional data and aligned with population needs and international standards—offers a strategic opportunity to strengthen the coherence, efficiency, and comparability of the national health system. Such reform is essential not only to improve population functioning and health outcomes, but also to sustain economic productivity and social inclusion in an ageing society increasingly affected by chronic conditions and long-term disability.

A modern rehabilitation workforce should rest on three pillars: scientific evidence, global comparability, and interprofessional collaboration. Achieving this requires overcoming fragmented professional silos and aligning education, regulation, and service delivery with population needs, and WHO and EU frameworks. While the normative case for reform is strong, implementation will depend on political commitment, institutional coordination, and sustainable financing. The recommendations below (Table 4) are therefore prioritized, time-bound, and supported by feasibility considerations and international experience.

**Table 4:** Policy recommendations for strengthening the rehabilitation workforce in Italy.

	
Short-term (0–2 years)	
Priority 1: Establish a national multisectoral advisory committee to coordinate action across ministries and regions	Indicator: Committee established; number of meetings and policy outputs/year Feasibility: High. Low financial cost. Risk of limited authority or duplication with existing bodies Example: National rehabilitation coordination platforms in countries like Canada have improved policy coherence
Priority 2: Clarify professional profiles and scopes of practice through a national regulatory framework to reduce fragmentation	Indicator: Adoption of national framework; % of professions with updated legal scope definitions Feasibility: Moderate. Requires legislative action. Potential resistance from established professional groups, and risk of national-regional fragmentation Example: Countries such as the UK and the Netherlands have implemented competency-based scope definitions to improve workforce efficiency
Priority 3: Integrate rehabilitation workforce data into national information systems	Indicator: Inclusion of rehabilitation indicators in national health information systems Feasibility: Moderate–high. Builds on existing systems. Risk of regional variability and interoperability challenges Example: Inclusion of rehabilitation workforce data in the National Health Information System in Spain^1^
Medium-term (3–5 years)	
Priority 1: Implement need-based workforce planning	Indicator: National workforce evaluation reports using standardized tools Feasibility: Moderate. Requires technical expertise and resource reallocation Example: Australia has applied needs-based workforce planning in allied health sectors
Priority 2: Integrate rehabilitation into primary care	Indicator: Number of primary care settings delivering rehabilitation services Feasibility: Moderate. Requires workforce redistribution and modification of existing hospital-centric funding models Example: Spain^2^ and the UK have integrated rehabilitation services within primary care
Priority 3: Align education and professional standards with international frameworks	Indicator: Number of programs aligned with international standards Feasibility: Moderate. Requires coordination between academic and regulatory institutions Example: The European Higher Education Area (Bologna Process) supports such harmonization
Priority 4: Develop workforce guidelines using GRADE methodology	Indicator: Number of guidelines developed using GRADE Feasibility: Moderate. Requires methodological expertise and consensus-building Example: Evidence-based workforce recommendations are increasingly used in WHO guideline development processes
Priority 5: Strengthen interprofessional education and practice	Indicator: % of universities implementing interprofessional education; student participation rates Feasibility: Moderate–high. Requires curriculum redesign and collaboration within universities Example: Sweden and Canada have successfully integrated interprofessional education in health curricula
Long-term (5–10 years)	
Priority 1: Link financing and accreditation to outcomes	Indicator: % of services using outcome-based financing/accreditation Feasibility: Low–moderate. Requires systemic reform and strong governance. Risk of political resistance and measurement complexity Example: Value-based healthcare models in the Netherlands and Germany incorporate outcome metrics

^1^Ministerio de Sanidad. Estadísticas y estudios de salud [Internet]. Madrid: Ministerio de Sanidad; [citado 31 mar 2026]. Disponible en: https://www.sanidad.gob.es/estadEstudios/portada/home.htm^2^Ministerio de Sanidad. Atención Primaria y Comunitaria: estrategias [Internet]. Madrid: Ministerio de Sanidad; [citado 31 mar 2026]. Disponible en: https://www.sanidad.gob.es/areas/calidadAsistencial/estrategias/atencionPrimaria/
